# A new insight into acute lymphoblastic leukemia in children: influences of changed intestinal microfloras

**DOI:** 10.1186/s12887-020-02192-9

**Published:** 2020-12-07

**Authors:** Xiaolin Gao, Ruixue Miao, Yiping Zhu, Chao Lin, Xue Yang, Ruizhen Jia, Kuang Linghan, Chaomin Wan, Jianjun Deng

**Affiliations:** 1 Department of Paediatrics, Western Women’s and Children’s Research Institute, West China University Second Hospital, Sichuan University, Number 20, 3rd Section, People’s South Road, Chengdu, 610041 Sichuan Province China; 2 Key Laboratory of Birth Defects and Related Diseases of Women and Children, (Sichuan University), Ministry of Education, Chengdu, 610041 Sichuan China; 3 Open Laboratory, West China Institute for Women’s and Children’s Health, Chengdu, 610041 Sichuan China; 4 Group of bacterial biology, Department of Laboratory Medicine, Sichuan university west China second hospital, Chengdu, 610041 Sichuan China

**Keywords:** Acute lymphoblastic leukemia, Children, Intestinal microfloras, 16S rDNA sequencing

## Abstract

**Background:**

Previous studies have shown that changes in intestinal microfloras are associated with both gastrointestinal (GI) and non-GI tumors. It is not clear whether there is an association between GI microflora changes and hematological malignancies.

**Methods:**

In the current study, we used 16S rDNA gene sequencing techniques to profile the GI microbiome in children with lymphoblastic leukemia (ALL, *n* = 18) and matched healthy control (*n* = 18). Using multiple specialized software [Heatmap, Principal coordinates analysis (PCoA), Claster and Metastates], we analyzed the sequencing data for microfloral species classification, abundance and diversity.

**Results:**

A total of 27 genera between the ALL and control groups (*FDR* ≤ 0.05 and/or *P* ≤ 0.05) showed significantly different abundance between ALL patients and healthy controls: 12 of them were predominant in healthy group and other 15 species were significantly higher in ALL group. In addition, we compared the abundance and diversity of microfloral species in ALL patients prior to and during remission stage after chemotherapy, and no significant difference was detected.

**Conclusions:**

Compared to healthy controls, ALL patient showed significant changes of GI microfloras. Further explorations of the intestinal micro-ecology in ALL patients may provide important information to understand relationship between microfloras and ALL.

## Background

As the most common malignancy in children, leukemia accounts for about 1/4 to 1/3 of the total incidence of malignancies in children [1]. Acute lymphoblastic leukemia (ALL) accounts for 75% of all types of leukemias, and represents the most common type of leukemia [2]. ALL is known to have high morbidity and mortality, which are increased year by year. ALL poses a great threat to children’s health, and has been widely concerned all over the world [3, 4]. The etiology of and pathogenesis underlying ALL in children remains not conclusive, and the widely accepted concept currently is that genetic susceptibility and environmental influences are two key factors that affect the development and progression of leukemia [5, 6].

Previous studies have shown that changes in intestinal microfloras or their dysfunction were closely associated with human health and the development and progression of diseases [7, 8]. Our recent studies have demonstrated that changes in intestinal microfloras were correlated with a number of diseases, including nonalcoholic fatty liver disease, obesity, allergic purpura and diarrhea [9, 10]. Therefore, we are interested in whether the intestinal microfloras are changed in children with leukemia and whether the chemotherapeutic agents affect them. In the current study, we explored the intestinal microfloras in children with ALL in comparison to age- and sex-matched healthy controls using the 16S rDNA high-throughput sequencing technique. In this study, the results of α diversity, β diversity and principal coordinates analysis (PCoA) of the intestinal flora were compared between the two groups, with a simultaneous analysis of the changes of intestinal floras in children with ALL before and after chemotherapy. Our findings from the current study provided new insight on ALL pathogenesis, which either results in the changes of intestinal microfloras or its development/progression clarity an involvement of the change of microbiome.

## Methods

### Study design and subjects

This study was reviewed and filed by the Ethical Committee of West China Second University, all methods were performed in accordance with the relevant guidelines and regulations, and written informed consent was obtained from the guardians of the subjects (NO.20170508).

Patients were included in the ALL group if they (1) were initially diagnosed with ALL in children (See [11] for diagnostic criteria) during the time period between September and December, 2016 in West China Second University Hospital; (2) had not been administered with chemotherapeutic drugs; (3) had not received antibiotics and microbiological agents within the last 2 weeks; (4) were void of additional severe organ diseases, such as the liver and the kidneys; and (5) informed consent was signed and obtained from the subjects and/or their guardians.

The standard VDLP protocol by the Chinese Children’s Leukemia Group (CCLG)-ALL2017 was adopted as the treatment regimen for children with ALL until the clinical remission stage (bone marrow reexamination is usually performed after 33 days). This regimen consist of vincristine (Oncovin, VCR) at 1.5 mg/m2 once weekly through intravenous injection, Daunorubicin (DNR) at 25 mg/m2 once daily through intravenous injection, L-asparaginase (L-Asp) at 6000 U/m2 once every other day through intravenous injection or intramuscular injection, and Pegasa ginseng (Peg-Asp) at 2000 U/m2 through intramuscular injection. Adjustments in treatment regimens and symptomatic treatment could be given in response to special circumstances.

Those included in the control group were healthy children who underwent physical examinations in our hospital during the same period of recruitment for ALL patients. Informed consent was signed and obtained from healthy subjects and/or their guardians. The basic data of the subjects such as age, sex, height (m) and body weight (kg) were recorded for both ALL and control groups.

### Preparation of fecal samples and 16S rDNA high-throughput sequencing

Using sterile closed fecal boxes, fresh feces (5 g) expelled within 2 h were collected from healthy children, ALL children upon ALL diagnosis, and ALL children before treatment and after chemotherapy with achievement of clinical remission. Fecal samples were quickly placed in ultra-low temperature freezers (− 80 °C) for further processing and testing.

Fecal DNA was extracted using QIAamp DNA Stool Mini Procedure Fecal Extraction DNA Kit (QIAGEN, Germany). The concentration and purity of DNA were measured using UV-Vis spectrophotometer. Qualified DNA samples were sent to BGI Co. under refrigeration conditions for 16S rDNA V3-V4 hypervariable region PCR amplification, library construction, and Illumina Hiseq 2000 16S rDNA high-throughput sequencing.

### Bioinformatics analysis

The raw data for the 16S rDNA high-throughput sequencing were subjected to the quality analysis using the designated software, ensuring only data meeting the quality criteria for sequencing depth, coverage and uniformity were used for the further analysis for species classification, abundance analysis and diversity analysis (BGI Tech Co.). Analysis of high-throughput sequencing results was completed by entrusting BGI Tech Co. Ltd. using software such as Heatmap, PCoA, Claster, and Metastates.

Species classification was performed by comparing the Ribosomal Database Project (RDP) 16 s rDNA database with the Operational taxonomic units (OTUs) of the RDP classifier (v2.2 https://rdp.cme.msu.edu/classifier/classifier.jsp), and relative abundance of species at both the phylum and genus levels were also compared. The difference in the abundance of microfloras between the ALL group and the control group was measured by means of the rank sum test, and the significance of the difference was evaluated using False Discovery Rate (FDR) [12, 13].

αdiversity reflects the species diversity and abundance of microorganisms in a single specimen, including Chao index, ACE index, Shannon index and Simpson index. Among them, Chao index and ACE index are the indexes for calculating the abundance of flora, and Shannon index and Simpson index are the indexes for calculating flora diversity. Their high values indicate the high abundance and diversity of the flora samples. For the results of the inter-group comparison of α diversity index, continuous variables are expressed as mean ± standard deviation (SD), the means and SDs of α diversity are calculated in each group. If *P* < 0.05, the difference between groups is statistically significant, that is, there is a difference in species diversity between the obesity group and the control group, and the species of the intestinal flora in the control group is richer. The boxplot of α diversity can show the difference in α diversity between the groups more intuitively. The boxplot can present 5 statistics (minimum, first quartile, median, third median and maximum, as well as 5 lines from bottom to top), with outliers marked with “°” [12–14].

ACE formula:

$$ {S}_{ACE}\left\{\begin{array}{c}{S}_{abund}+\frac{s_{rare}}{C_{ACE}}+\frac{n_1}{C_{ACE}}, for\ {\gamma}_{ACE}^2<0.08\\ {}{S}_{abund}+\frac{s_{rare}}{C_{ACE}}+\frac{n_1}{C_{ACE}}, for\ {\gamma}_{ACE}^2\geqq 0.08\end{array}\right. $$

Chao formula:

$$ {S}_{chao1}={S}_{obs}+\frac{n_1\left({n}_1-1\right)}{2\left({n}_2+1\right)} $$

Shannon formula:

$$ {H}_{shannon}=-{\sum}_{i=1}^{S_{obs}}\frac{n_i}{N}\mathit{\ln}\frac{n_i}{N} $$

Simpson formula:

$$ {\mathrm{D}}_{\mathrm{simpson}}=\frac{\sum_{i=1}^{S_{obs}}{n}_i\left({n}_i-1\right)}{N\left(N-1\right)} $$

βdiversity refers to the range of changes in community composition, which describes the changes of species composition in time and space. β diversity analysis can be realized by multivariate statistical methods including Principal coordinates analysis (PCoA) and clustering analysis, which can directly present the similarity and difference of the complex intestinal flora, and can be used to compare the difference in species diversity between a pair of samples. PCoA is one of the most commonly used unconstrained sorting methods based on linear model. That is, without considering the influence of environmental factors or any foresight to the samples, the internal structure of the samples is observed without bias, and one or more potential variables (i.e. principal component, PC) are obtained, which can used to best predict the values of all species, so as to achieve dimension reduction. In linear model, the score of samples is a linear combination of species score. In the study on microbial composition and structure, OTU or evolutionary types are always used to represent species information. In relevant study on the intestinal flora, PCoA is widely applied to compare the composition of different intestinal floras [12–14].

### Statistical analysis

The data were analyzed and compared using the SPSS package. The normally distributed measurement data were expressed as mean ± standard deviation ($\overline{x}$ ± s). The paired *t-*test was employed for comparison of mean numbers of randomly designed samples before and after the treatment. *P* ≤ 0.05 indicated statistical significance.

### Availability of materials and data statement

Materials, data and associated protocols are promptly available to readers without undue qualifications in material transfer agreements.

## Results

### Subjects

A total of 36 subjects were enrolled in the current study. ALL group and the control group each have 18 subjects (Table 1). There were no significant differences between the two groups in terms of their age, sex and height (*P* > 0.05).

**Table 1:** Basic information for subjects in the current study($\overline{x}$ ± s)

	ALL (*n* = 18)	Control (*n* = 18)
**Male (n)**	9	10
**Female (n)**	9	8
**Age (y)**	6.31 ± 1.25	5.89 ± 2.19
**Height (m)**	1.17 ± 0.29	1.18 ± 0.38
**Weight (kg)**	21.64 ± 2.07	20.19 ± 2.37

### Basic sequencing information

A total of 611,034 effective 16S rRNA sequences were obtained upon the completion of the sequencing on the Illumina Hiseq platform. Using the manufacture software analysis for splicing quality control, it was found that the largest and smallest sequence numbers were 27,179 and 6556, respectively. The number of valid sequences were 17,000.26 ± 1497.46 in the ALL group and 17,890.56 ± 2811.56 in the control group, which was significantly different (*P <* 0.05). A total of 2913 operational taxonomic units were obtained by clustering sequences with 97% similarity, with a significant distribution in the ALL group (61.41 ± 2.38) and in the control group (103.83 ± 2.46) (*P <* 0.05).

### Analysis of complexity of intestinal microfloras

#### The α diversity

**Fig. 1:**
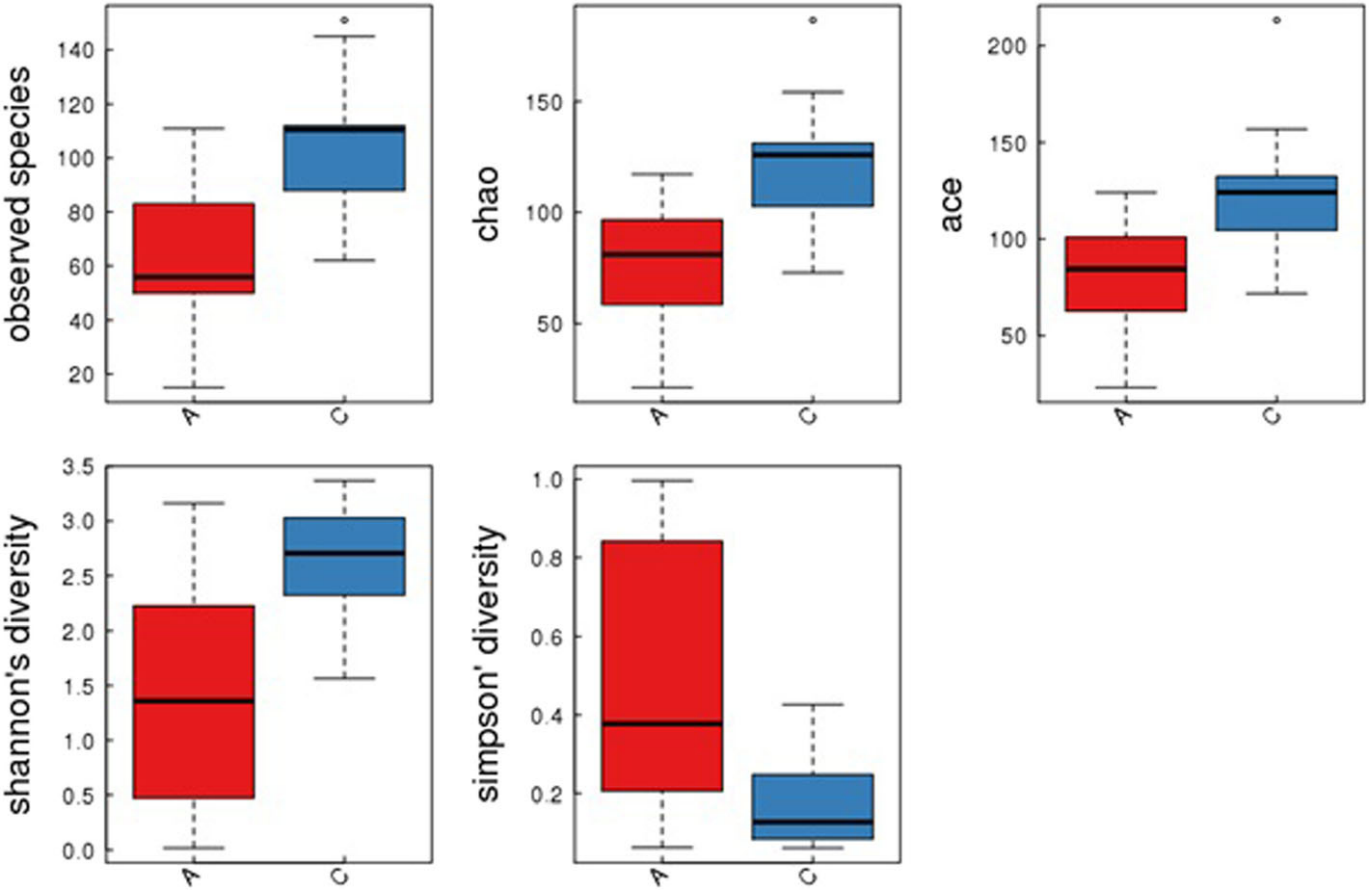
Box-plot of α diversity of the ALL group and the control group. Red boxes represent the ALL group, and blue boxes represent the control group. The Chao index (the ALL group 74.01 ± 28.81, the control group 121.70 ± 27.07, *P* < 0.00), the ACE index (the ALL group 82.40 ± 27.59, the control group 122.76 ± 30.53, *P* < 0.00), the Shannon index (the ALL group 1.42 ± 1.04, the control group 2.63 ± 0.53, *P* < 0.00) and the Simpson index (the ALL group 0.49 ± 0.34, the control group 0.17 ± 0.11, *P* < 0.00) were calculated for the comparison of α diversity

The dilution curve showed an inflection point within 1000 and gradually plateaued. Using the designated software, data meeting the quality criteria for sequencing depth, coverage and uniformity were included for further analysis of species abundance and and diversity. The Observed species index, Chao index, ACE index, Shannon index and Simpson index were calculated for the comparison of α diversity. As shown in Fig. 1, all of these indexes showed statistical significance (*P* < 0.05) between between the ALL group and the control group.

#### The β diversity

**Fig. 2:**
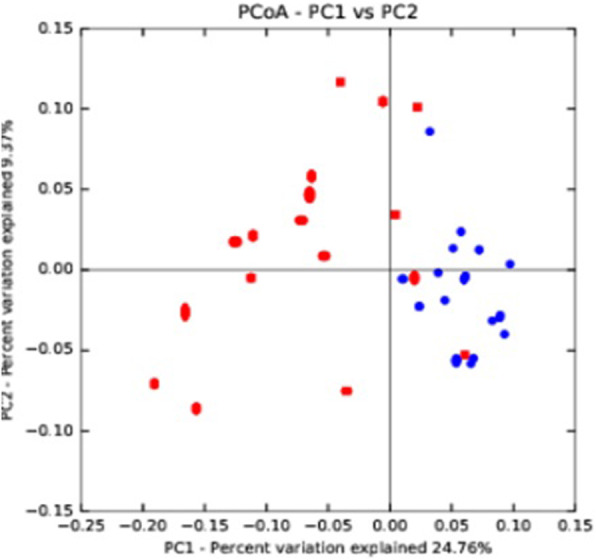
Principal coordinates analysis (PCoA) of the ALL group and the control group based on Operational taxonomic units (OTU) abundance. Red points indicate the ALL group, and blue points represent the control group

Figure 2 showed a PCoA chart, which includes all of 36 sequenced samples (red dot = ALL group, and blue point = control group). PC1 was the first principle coordinate, representing 24.76% of the total microfloras; the vertical axis was PC2, accounting for 9.37% of the total microfloras. While most of the red dots clustered on the left of the graph, most of blue dots clustered on the right side of the graph, indicating a clear separation of ALL and healthy control group in term of β diversity.

### Species abundance analysis in ALL patients and healthy controls

**Fig. 3:**
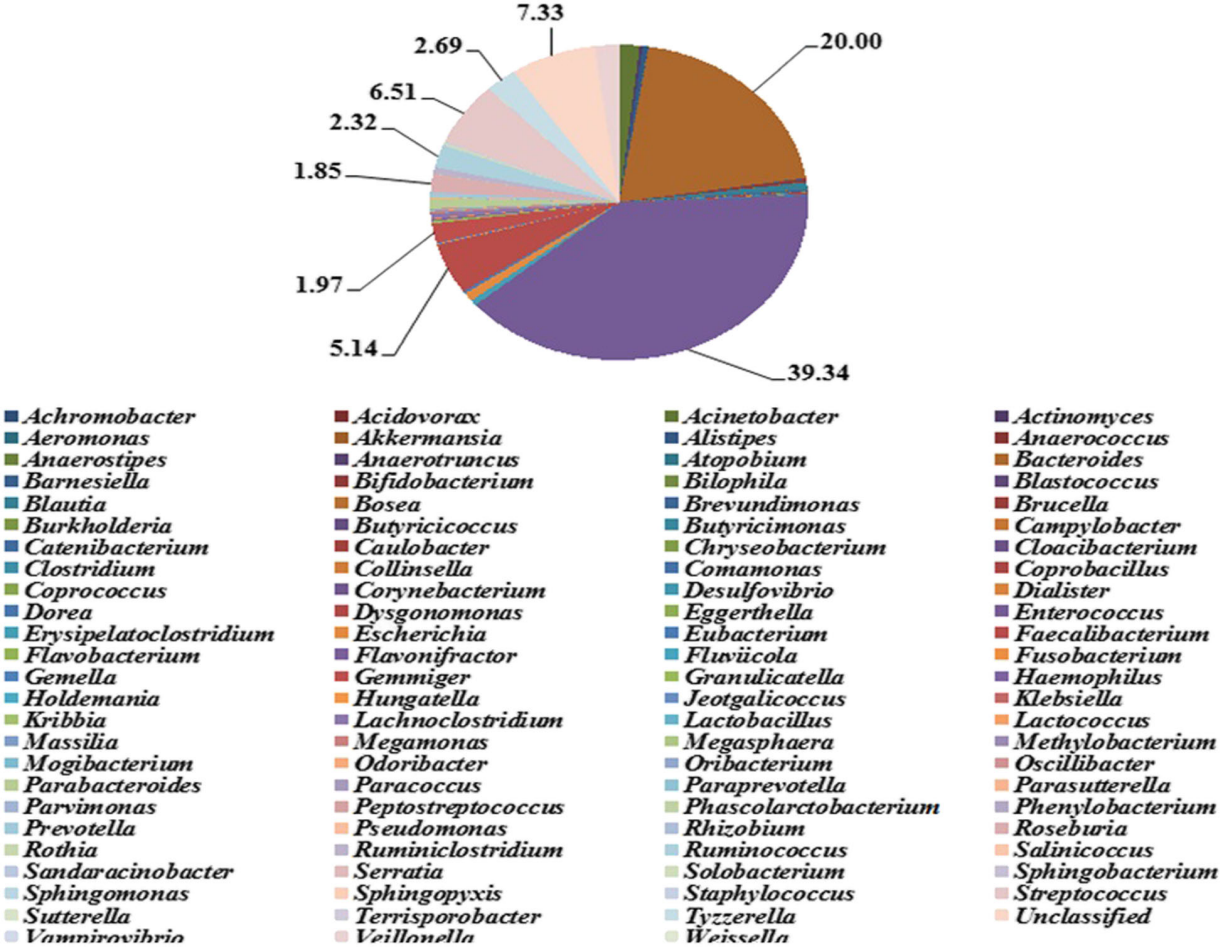
Relative abundance of species at the classification level of Genus in the ALL group. A total of 99 genera were found at the classification level of genus. Among the 99 genera of bacteria in the ALL group, *Enterococcus* was the predominant genus (39.34%, in purple), *Bacteroides* was 20% in brown, *Unclassified* was 7.33% in shallow orange, *Streptococcus* was 6.51% in shallow red, *Faecalibacterium* was 5.14% in red, and so on. Among these genera, the dominant species in ALL patients and healthy controls showed significant differences, a total of 12 species including *Acinetobacter, Actinomyces, Bosea, Brevundimonas, Enterococcus, Megasphaera, Oribacterium, Rhizobium, Ruminiclostridium, Sphingomonas, Tyzzerella and Veillonella* were more abundant than in the control group

**Fig. 4:**
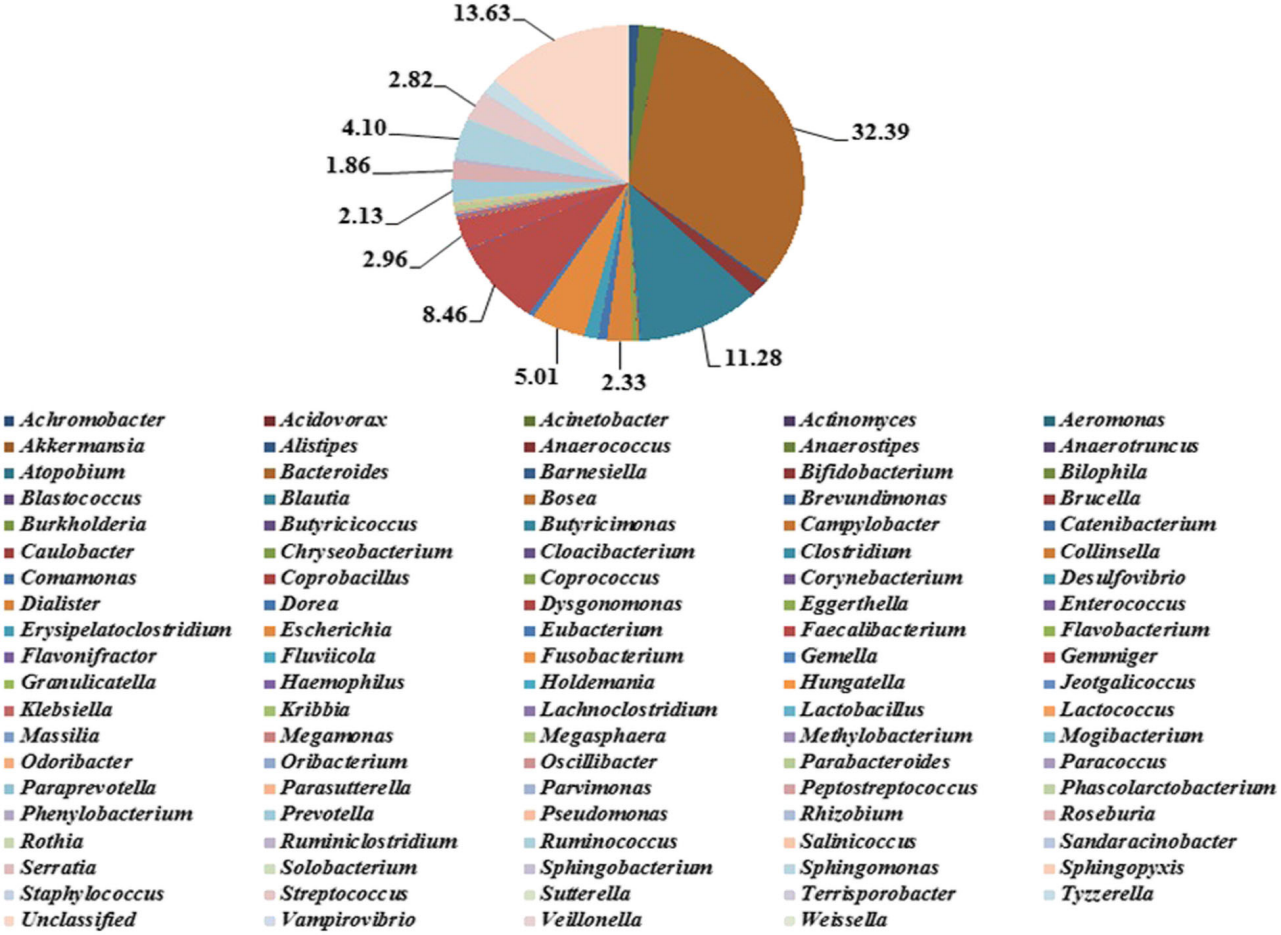
Relative abundance of species at the classification level of Genus in the control group. A total of 99 genera were found at the classification level of genus. Among the 99 genera of bacteria in the control group, *Bacteroides* was the predominant genus in the control group (32.39%, in brown), *Unclassified* was 13.63% in shallow orange, *Blautia* was 11.28% in blue, *Faecalibacterium* was 8.46% in red, *Escherichia* was 5.01% in orange, *Ruminococcus* was 4.10% in shallow blue, and so on. Among these genera in contrast, a total of 15 species, including *Anaerostipes, Bifidobacterium, Blautia, Collinsella, Dialister, Dorea, Erysipelatoclostridium, Faecalibacterium, Lactobacillus, Oscillibacter, Prevotella, Roseburia, Ruminococcus, Terrisporobacter* showed a higher abundance in the ALL group

A total of 99 genera were found at the classification level of genus, showing that the overall microfloras in the control group were more abundant than in the ALL group (Figs. 3 and 4). Among the 99 genera of bacteria, *Enterococcus* was the predominant genus in the ALL group (39.34%), which was significantly different from that in the control group [*False Discovery Rate* (*FDR)* < 0.05, *P* < 0.05]. *Bacteroides* was the predominant genus in the control group (32.39%), while the difference was not statistically significant as compared with the ALL group (*FDR* > 0.05). Significant differences were present in a total of 27 genera between the ALL and control groups (*FDR* ≤ 0.05, *P* ≤ 0.05). Among these genera, the dominant species in ALL patients and healthy controls showed significant differences, which are summarized in Table 2 Briefly, a total of 12 species including *Acinetobacter, Actinomyces, Bosea, Brevundimonas, Enterococcus, Megasphaera, Oribacterium, Rhizobium, Ruminiclostridium, Sphingomonas, Tyzzerella* and *Veillonella* were more abundant than in the control group. In contrast, a total of 15 species, including *Anaerostipes, Bifidobacterium, Blautia, Collinsella, Dialister, Dorea, Erysipelatoclostridium, Faecalibacterium, Lactobacillus, Oscillibacter, Prevotella, Roseburia, Ruminococcus, Terrisporobacter* showed a higher abundance in the ALL group.

**Table 2:** The dominant species in the ALL and control groups

Species	ALL (%)	Control (%)	*P*-value	FDR
Dominant species in both groups				
Bacteroides	20.00	32.39	0.02934	0.08068
Species more dominant in ALL group				
Acinetobacter	**1.72**	0.01	0.004574	0.026637
Actinomyces	**0.24**	0.01	0.000555	0.006868
Bosea	**0.02**	0.00	0.007178	0.035781
Brevundimonas	**0.14**	0.01	0.008895	0.035781
Enterococcus	**39.34**	0.02	2.50E-05	0.00082
Megasphaera	**0.02**	0.00	0.003155	0.020823
Oribacterium	**0.13**	0.00	0.004077	0.025226
Sphingomonas	**0.10**	0.02	0.003049	0.020823
Rhizobium	**0.09**	0.01	0.008647	0.03578
Ruminiclostridium	**0.67**	0.37	0.002964	0.02082
Tyzzerella	**2.69**	1.50	0.006264	0.03445
Veillonella	**2.08**	0.09	0.009397	0.035781
Species more dominant in control group				
Anaerostipes	0.03	**2.25**	1.00E-06	0.000099
Bifidobacterium	0.36	**1.61**	0.000353	0.006492
Blautia	0.70	**11.28**	4.00E-06	0.000198
Collinsella	0.00	**0.16**	0.002459	0.020287
Dialister	0.02	**2.33**	0.008983	0.035781
Dorea	0.21	**0.83**	0.000459	0.006492
Erysipelatoclostridium	0.57	**1.25**	0.00853	0.035781
Faecalibacterium	5.14	**8.46**	0.00226	0.020287
Lactobacillus	0.02	**0.08**	0.002151	0.020287
Oscillibacter	0.07	**0.15**	0.000351	0.006492
Prevotella	0.38	**2.13**	0.013201	0.048404
Roseburia	1.85	**1.86**	0.007937	0.035781
Ruminococcus	2.32	**4.10**	0.000406	0.006492
Terrisporobacter	0.03	**0.15**	0.000846	0.009306
Unclassified	7.33	**13.63**	0.009265	0.035781

### Comparison between intestinal microfloras in the ALL group before chemotherapy and during the clinical remission stage

Neither the α diversity (Table 1) nor β diversity (Fig. 1) showed statistical difference in ALL patient before and after chemotherapy (*P* > 0.05). In addition, there was no significant difference in the abundance of intestinal microfloras between them (*FDR* > 0.05, *P* > 0.05). Real-time PCR was also performed for two representative species, (*Bifidobacterium* (*B*) and *Escherichia coli* (*E*)]. As showed in (Supplementary Fig. 1), while *E coli* did not show difference, *Bifidobacterium* showed a significant decrease. It was suggested that quantification of for overall microbiome using sequencing method may differ from the quantification of individual species using real-time PCR method.

Additional analysis for the α diversity and β diversity between ALL in remission stage (after chemotherapy) and healthy control did not detect the difference (Table 2, Figs. 3 and 4, respectively).

## Discussion

It is reported that 20% of cancers in the world are associated with microbes [15]. Gastrointestinal microbes can affect the integrity of DNAs and immune regulation and promote the development and progression of gastrointestinal tumors by inducing inflammations, increasing cell proliferation, changing kinetics of stem cells and producing metabolites, such as butyric acid [16]. A number of studies have shown that changes in intestinal microfloras are not only associated with digestive system tumors such as gastric cancer [17], liver cancer [18] and colon cancer [19], but also correlated with non-gastrointestinal tumors, such as breast cancer [20]. However, the study for the change of intestinal microfloras is still in the beginning stage, and only one publication has been found in the literature [21].

Our findings showed that the number of sequences, the number of OTU and the abundance of intestinal microfloras in the ALL group were significantly lower than in the healthy control group. In addition, between the ALL and healthy control groups were also significantly in the structures for the dominance of intestinal microfloras. Some studies have shown that well-balanced microbiome in the gut plays important roles in human health through the involvement of biological antagonism, immune regulation and nutrition. However, the study for its change in ALL patients is still in the beginning stage. We have very limited information in understanding their roles in this disease. Based on the previous findings of microbiome changes in other diseases, we hypothesized that the difference of the dominant species in ALL from these in healthy controls could be the resultant change from ALL disease or a factor that may facilitate the development and/or progression of ALL. On one hand, the disruption of immune system in ALL may create an environment favorable for certain gut microfloral growth. On the other hand, acquisition of dominance of certain microfloral growth due to other undefined reasons may comprise immunosurveillance and allow malignant clonal expansion for ALL. The finding of significant difference of microflora between the ALL and healthy control group highly motivated us to further investigate the implication of microfloral change in ALL in the future.

Our study revealed that there was no significant difference in the intestinal microfloras in patients in the ALL group before chemotherapy and during the clinical remission stage, suggesting that the chemotherapeutic drugs for ALL had no significant effects on intestinal microfloras. Our findings were different from some reports. van Vliet MJ et al. reported that during chemotherapy in patients with acute myeloid leukemia, the total number of bacteria in the fecal samples detected and analyzed using polymerase chain reaction-denaturing gradient gel electrophoresis fingerprint (DGGE) was 100-fold fewer than that in the healthy controls, and that the numbers of anaerobic bacteria and the potentially pathogenic aerobic enterococci were decreased and increased, respectively [22]. Huang Y et al. reported that detection of the feces of ALL children on high-dose chemotherapy using such methods as real-time PCR showed that the number of *lactobacilli* and *Escherichia coli* after chemotherapy was significantly lower as compared with that in the control group [23].

With regard to the impact of chemotherapy on microflora in ALL patient, while it generally thought that chemotherapy changed the diversity of microbiome, our current study did not find such impact, which is consistent with two previous publications. One study conducted by Nyhlén A [24], showed that most of patient showed stable intestinal microflora during chemotherapy. In addition, Rajagopala SV [21] also used the sequencing method (similar methods used in our current study) to measure the abundance of gut microflora in ALL patients before and after chemotherapy. It was found that the microbiome diversity was influenced by a variety of factors including the antibiotics and steroids in the combination of chemotherapy. In our study, the studied subjects were all free of antibiotics for 2 weeks before the sample collection. This may explain why our patients did not show the difference before and after chemotherapy. In addition, data derived from real-time PCR quantification for specific species showed the difference in patients before and after chemotherapy. It appears that the different detection methods may also give rise to different results. In conclusion, the diversity of patient populations with different treatment regimen (especially concurrent administration of antibiotics and immunoregulatory drugs) as well as the different detection methods may collectively contribute to the controversial impact of chemotherapy on gut microflora.

Real-time fluorescent quantitative PCR is currently widely used for nucleic acid detection. Real-time PCR possess many advantages, such as high sensitivity, accuracy, and great reproducibility. However, based on our previous experience to use this technique to examine the intestinal microbiome [9, 10], it is very costly as well as labor- and time-consuming, because it is necessary to design specific primer pair for each species. In addition to designing hundreds of such primer pairs, a standard curve need to be established with already known samples for each species. As a result, a study with real-time PCR will have to focus on certain species with limited number of species to examine. Therefore, study with real-time PCR will have limited value in understanding the overall change of gut microbiome. Fortunately, the high through sequencing techniques become available and it allows us to sequence millions of DNA molecule at the same time and provide a data pool to cover the entire microbiome in the gut. Because this sequencing method preserve the integrity of whole microbiome and calculate the amount of different species according to the number of matched sequences to a specific species [22, 23], the measurement of abundance of the species using a more sophisticated algorithm. Due to these unique capacities of sequencing method, we believe that it is superior to real time PCR. With regard to the discrepancy between the results from the two methods, we think that the sequencing method provides more accurate estimation of microfloral distribution.

## Conclusions

In conclusion, in the past decade, the relationship between intestinal microfloras and diseases has become the frontier and hot research topic in the field of microecology. In the current study, we found that the dominant intestinal microfloras in children with ALL were significantly different when compared to the healthy control. Furthermore, chemotherapeutic drugs have no significant effects on the intestinal microfloras of children with ALL. These results are very interesting, which provide reference for the clinical treatment of ALL. The intestinal microfloras are large in number and complex in function, and the specific mechanisms of action and pathways, explorations of the intestinal micro-ecology in ALL patients may provide us important information to understand relationship between microfloras and this disease

## Supplementary information

**Additional file 1.**

## Data Availability

The authors declare the availability of data and material.

## Abbreviations

GI: Gastrointestinal
ALL: Acute lymphoblastic leukemia
PCoA: Principal coordinates analysis
CCLG: Chinese Children’s Leukemia Group
RDP: Ribosomal Database Project
OTU: Operational taxonomic unit
FDR: False Discovery Rate

## References

[1] Kaatsch P (2010). Epidemiology of children cancer. Cancer Treat Rev.

[2] Hashemizadeh H, Boroumand H, Noori R, Darabian M (2013). Socioeconomic status and other characteristics in childhood leukemia. Iran J Ped Hematol Oncol.

[3] Tong N, Xu B, Shi D, Du M, Li X, Sheng XJ (2014). Hsa-miR-196a2 polymorphism increases the risk of acute lymphoblastic leukemia in Chinese children. Mutat Res.

[4] SHAQ Multicenter Study Group of Children’s Acute Lymphoblastic Leukemia Research (2013). Multi-center trial based on SCMC-ALL-2005 for children’s acute lymphoblastic leukemia. Zhonghua Er Ke Za Zhi.

[5] Zhao L, Liu X, Wang C, Yan KK, Lin XJ, Li S (2014). Magnetic fields exposure and childhood leukemia risk: a meta-analysis based on 11,699 cases and 13,194 controls. Leuk Res.

[6] Gao Y, Zhang Y, Kamijima M, Sakai K, Khalequzzaman M, Nakajima T (2014). Quantitative assessments of indoor air pollution and the risk of childhood acute leukemia in Shanghai. Environ Pollut.

[7] Doycheva I, Leise MD, Watt KD (2016). The intestinal microbiome and the liver transplant recipient: what we know and what we need to know. Transplantation..

[8] Li J, Butcher J, Mack D, Stintzi A (2015). Functional impacts of the intestinal microbiome in the pathogenesis of inflammatory bowel disease. Inflamm Bowel Dis.

[9] Xiaolin G, Yu Z, Yang W, Liu GJ, Wan CM. Efficacy of probiotics in nonalcoholic fatty liver disease in adult and children: A meta-analysis of randomized controlled trials. Hepatol Res. 2016. 10.1111/hepr.12671. 10.1111/hepr.12671 26866817

[10] Xiaolin G, Ruizhen J, Liang X, Linghan K, Ling F, Chaomin W (2015). Obesity in school-aged children and its correlation with Gut E.coli and Bifidobacteria:a case-control study. BMC Pediatr.

[11] Colin D. Rudolph, George E. Lister, Lewis R. First. Rudolph’s Pediatrics 22E. 2010;1590–1596.

[12] David PC, Nanette JP, Michelle RM (2018). Molecular Biology. Academic Cell.

[13] Lesk A (2019). Introduction to bioinformatics. Oxford Univ Press U S A.

[14] Stoof-Leichsenring KR, Dulias K, Biskaborn BK (2020). Lake-depth related pattern of genetic and morphological diatom diversity in boreal Lake Bolshoe Toko, Eastern Siberia. PLoS One.

[15] Pevsner-Fischer M, Tuganbaev T, Meijer M, Zhang SH, Zeng ZR, Chen MH (2016). Role of the microbiome in non-gastrointestinal cancers. World J Clin Oncol.

[16] Abreu MT, Peek RM (2014). Gastrointestinal malignancy and the microbiome. Gastroenterology..

[17] Lertpiriyapong K, Whary MT, Muthupalani S, Lofgren Jennifer L, Gamazon Eric R, Feng Y (2014). Gastric colonisation with a restricted commensal microbiota replicates the promotion of neoplastic lesions by diverse intestinal microbiota in the helicobacter pylori INS-GAS mouse model of gastric carcinogenesis. Gut..

[18] Yoshimoto S, Loo TM, Tarashi K, Kanda H, Sato S, Oyadomari S (2013). Obesity-induced gut microbial metabolite promotes liver cancer through senescence secretome. Nature.

[19] Fehlbaum S, Chassard C, Haug MC, Fourmestraux C, Derrien M, Lacroix C (2015). Design and investigation of PolyFermS in vitro continuous fermentation models inoculated with immobilized fecal microbiota mimicking the elderly colon. PLoS One.

[20] Kwa M, Plottel CS, Blaser MJ, Adams S (2016). The intestinal microbiome and estrogen receptor-positive female breast cancer. J Natl Cancer Inst.

[21] Rajagopala SV, Yooseph S, Harkins DM, Moncera KJ, Zabokrtsky KB, Torralba MG, Tovchigrechko A, Highlander SK, Pieper R, Sender L, Nelson KE (2016). Gastrointestinal microbial populations can distinguish pediatric and adolescent acute lymphoblastic leukemia (ALL) at the time of disease diagnosis. BMC Genomics.

[22] van Vliet MJ, Tissing WJ, Dun CA, Meessen NEL, Kamps WA, de Bont ESJM (2009). Chemotherapy treatment in pediatric patients with acute myeloid leukemia receiving antimicrobial prophylaxis leads to a relative increase of colonization with potentially pathogenic bacteria in the gut. Clin Infect Dis.

[23] Huang Y, Yang W, Liu H, Duan J, Zhang Y, Liu M (2012). Effect of high-dose methotrexate chemotherapy on intestinal Bifidobacteria, Lactobacillus and *Escherichia coli* in children with acute lymphoblastic leukemia. Exp Biol Med (Maywood).

[24] Nyhlén A, Ljungberg B, Nilsson-Ehle I, Nord CE (2002). Impact of combinations of antineoplastic drugs on intestinal microflora in 9 patients with leukaemia. Scand J Infect Dis.

