# Supplementary figures and images for: A new insight into acute lymphoblastic leukemia in children: influences of changed intestinal microfloras
Source: BMC Pediatr. 2020 Dec 7;20:290. doi: 10.1186/s12887-020-02192-9 (PMC7646195; doi:10.1186/s12887-020-02192-9)

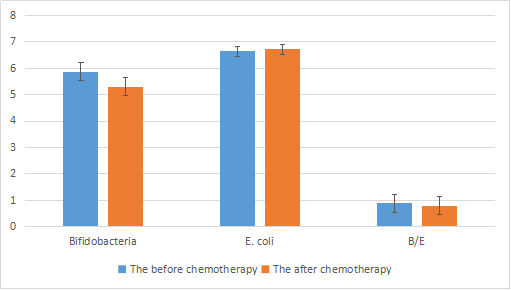

Supplement: Supplementary file 1 — Additional file 1. [file 12887_2020_2192_MOESM1_ESM.png]
